# Lessons Learned From Transition of an In-Person to a Virtual Randomized Controlled Trial for Weight and Fitness Concerns in Active-Duty Service Members: Survey Study

**DOI:** 10.2196/37797

**Published:** 2022-11-10

**Authors:** Niloofar Afari, Natalie M Yarish, Jennalee S Wooldridge, Karla Materna, Jeffrey Hernandez, Brian H Blanco, Angela L Camodeca, Joshua J Peters, Matthew S Herbert

**Affiliations:** 1 Department of Psychiatry University of California, San Diego La Jolla, CA United States; 2 Veterans Affairs San Diego Healthcare System San Diego, CA United States; 3 Veterans Affairs Center of Excellence for Stress and Mental Health San Diego, CA United States; 4 Navy Medicine Readiness and Training Command San Diego, CA United States

**Keywords:** virtualization, weight-loss intervention, lessons learned, military personnel, acceptance and commitment therapy

## Abstract

**Background:**

This paper describes and discusses the transition of and modifications to a weight management randomized controlled trial among active-duty military personnel from an in-person to a virtual format as a result of the COVID-19 pandemic. The original pragmatic cohort-randomized controlled trial was designed to compare the effectiveness of an 8-week group weight management program, ShipShape, to a version of ShipShape enhanced with acceptance and commitment therapy.

**Objective:**

The objective of our study was to assess potential differences between in-person and virtual participation in participants’ demographics, motivation, confidence, credibility, expectations, and satisfaction with the interventions; we also examined the pragmatics of the technology and participants’ experiences in virtual-format intervention groups.

**Methods:**

A total of 178 active-duty personnel who had failed or were at risk of failing their physical fitness assessment or were overweight or obese were enrolled in the study. In-person (n=149) and virtual (n=29) participants reported demographics, motivation, confidence, credibility, expectations, and satisfaction. Interventionists recorded attendance and participation in the group sessions. Independent-sample 2-tailed *t* tests and chi-square tests were used to compare the characteristics of the in-person and virtual participants. Pragmatics of the technology and participants’ experiences in the virtual format were assessed through surveys and open-ended questions.

**Results:**

Participants were 29.7 (SD 6.9) years old on average, 61.8% (110/178) female, and 59.6% (106/178) White and had an average BMI of 33.1 (SD 3.9) kg/m^2^. Participants were highly motivated to participate and confident in their ability to complete a weight management program. A total of 82.6% (147/178) of all participants attended 5 of the 8 sessions, and participation was rated as “excellent” by interventionists in both formats. The interventions were found to be credible and to have adequate expectations for effectiveness and high satisfaction in both formats. There were no differences between in-person and virtual participants in any of these metrics, other than interventionist-rated participation, for which virtual participants had significantly higher ratings (*P*<.001). Technical satisfaction with the virtual sessions was rated as “good” to “very good,” and participants were satisfied with the content of the virtual sessions. A word cloud of responses identified “mindfulness,” “helpful,” “different,” “food,” “binder,” and “class” as concepts the virtual participants found most useful about the program.

**Conclusions:**

Modifications made in response to the COVID-19 pandemic were successful, given the recruitment of active-duty personnel with similar demographic characteristics, attendance levels, and indicators of credibility, expectancy, and satisfaction in the virtual format and the in-person format. This successful transition provides support for the use of virtual or digital weight management interventions to increase accessibility and reach among highly mobile active-duty personnel.

**Trial Registration:**

ClinicalTrials.gov NCT03029507; https://clinicaltrials.gov/ct2/show/NCT03029507

## Introduction

To slow the spread of COVID-19, the World Health Organization declared a global pandemic and urged every country to take immediate and aggressive action on March 11, 2020 [1]. Stay-at-home orders were announced on March 19, 2020, that mandated all California residents to stay in their homes unless they needed to leave for essential work or activities. Like many other institutions, in-person research activities were halted, including our pragmatic randomized controlled trial (RCT) of a weight management program in active-duty military personnel that was funded by the National Institutes of Health (NIH) [2]. The RCT was designed to compare Navy’s ShipShape (SS) weight management program to an acceptance and commitment therapy (ACT)-enhanced SS program. Over 8 months, our research team transitioned the protocols and materials for this RCT and the intervention groups from an in-person to a virtual format. This paper describes and discusses this transition and identifies lessons learned to make recommendations relevant to RCTs and interventions with active-duty personnel.

The US Navy implemented the evidence-based SS program to address weight management and fitness among active-duty personnel who had failed or were at risk of failing the Physical Fitness Assessment (PFA) or were overweight or obese. Given the growing research that supports incorporating ACT—a transdiagnostic cognitive behavioral therapy designed to increase psychological flexibility [3]—into weight loss interventions [4-6], we obtained NIH funding in 2016 to conduct such a study with active-duty personnel. The primary aim of this RCT was to compare the effectiveness of the standard SS program and an ACT plus SS program on weight loss as a primary outcome and to examine several secondary outcomes among active-duty personnel at the Naval Medical Center San Diego (NMCSD), a large military treatment facility in southern California. Due to the pragmatic nature of the RCT, which required conducting the study in a real-world setting, the RCT was implemented in the NMCSD Health and Wellness Department. Study and clinic staff worked together collaboratively to carry out the study procedures and deliver the SS and ACT plus SS group interventions.

The pandemic-related restrictions initiated in March 2020 led to the temporary suspension of NMCSD Health and Wellness Department in-person activities, including the study groups. Soon thereafter, the Navy’s PFA requirements were suspended until at least June 2021, and NMCSD made the decision not to offer the SS program until then. The study team, in consultation with the NIH program officer and the study safety officer, determined that the study would continue through community recruitment and virtual study and intervention procedures. This decision was based on two rationales: (1) we anticipated that active-duty personnel, like many others, would continue to struggle with weight management through the pandemic, and (2) although there is growing evidence that technology-supported ACT can be effective in addressing health issues [7], including weight loss [8], we were not aware of any studies that had examined group-based virtual interventions for weight management among active-duty personnel. Further, examining the feasibility and successful implementation of a virtual study of active-duty personnel is particularly important given the limited number of RCTs of behavioral interventions with military personnel [9] and the well-known challenges of recruiting and retaining active-duty personnel for in-person studies [10].

This paper describes a series of modifications that were made to the study procedures and intervention protocols to transition the study from an in-person to a virtual format in the context of the COVID-19 pandemic. Additionally, we (1) assessed potential differences between in-person and virtual participants in demographics, motivation, confidence, credibility, expectations, and satisfaction with the interventions; and (2) examined the pragmatics of the technology and participants’ experiences in virtual-format intervention groups.

## Methods

### Original Study Overview

#### Background

The design, aims, procedures, and interventions of the original pragmatic cohort RCT trial have been described in detail elsewhere [2]. In brief, eligible participants included active-duty personnel between the ages of 18 to 69 years who had failed or were at risk of failing the PFA or were overweight or obese, were free of physical limitations that would prevent participation in physical fitness activities, and were not pregnant or planning to become pregnant in the next 6 months. Before the pandemic, in-person recruitment occurred primarily through referrals from the Navy’s Fitness Enhancement Program with the option to enroll in SS. Study staff met with interested participants after a Navy-facilitated orientation, briefly described the study, and asked them to complete a brief screening assessment. Eligible participants provided in-person consent after the orientation. If they consented, they were enrolled in the study and randomized to receive either an SS or ACT plus SS group intervention. A computer-generated randomization schedule was developed prior to the start of the data collection by the study statistician, and each cohort was randomly assigned to SS or ACT plus SS with equal probability.

Participants completed a battery of standardized, self-reported psychosocial measures and assessments of weight (as the primary outcome) and body fat percentage at week 1 (baseline), week 4 (midpoint), week 8 (posttreatment), a 3-month follow-up, and a 6-month follow-up. Baseline, midpoint, and posttreatment assessments were completed primarily in person, and measurements were taken by the SS coordinator or another member of the NMCSD Health and Wellness Department. The 3- and 6-month follow-up assessments were either scheduled to be in person or were completed remotely by mail or telephone, depending on the availability of the participant.

#### SS Intervention

The SS intervention was standard care at NMCSD and was based on guidelines from the Navy and Marine Corps Public Health Center [11]. Intervention topics included education about nutrition and physical activity; setting weight-loss goals; tracking eating habits; and initiating, monitoring, and maintaining physical activity levels. The group sessions were primarily led by the NMCSD SS coordinator (with 2 sessions led by an NMCSD social worker) and were held for 2 hours weekly over 8 weeks.

#### ACT Plus SS Intervention

The study team developed the ACT plus SS intervention by incorporating ACT principles and methods into the standard SS protocol. These concepts and strategies included (1) identifying thoughts, feelings, and bodily sensations surrounding weight loss efforts and overcoming limitations of previous efforts to control or eliminate negative thoughts or emotions, stress, or food cravings; (2) clarifying personal values that aligned with goals to improve health and quality of life; and (3) incorporating mindfulness exercises to increase present-moment awareness. Sessions were co-led by the NMCSD SS coordinator, who provided SS materials, and a study interventionist, who provided ACT content and experiential exercises. Sessions were held for 2 hours weekly over 8 weeks.

### Study Transition to Virtual Format

#### Outline

Table 1 provides an outline of modifications made to recruitment, screening, consent, assessment, and intervention procedures.

**Table 1:** Transition from in-person to virtual recruitment, screening, consent, assessment, and intervention.

Stage	In-person format	Virtual format
Recruitment	Referrals primarily from the Navy’s Fitness Enhancement Program. Recruits attended a ShipShape program orientation, and study staff explained the study.	The study was advertised through social media platforms, including Facebook, Instagram, and Twitter. Individuals in the San Diego community responded to the advertisements.
Screening	Study staff met with interested participants after a Navy-facilitated health and wellness orientation. Potential participants were screened immediately after orientation.	An online screening form asked potential participants questions about their active-duty status, overweight or obesity status, first name, phone number, and best time to call. Study staff conducted additional phone screening to determine the eligibility of interested participants, and then scheduled a virtual consent appointment.
Consent	Study staff reviewed the study procedures (including compensation, participation commitment, contacts, and study timelines) and provided the study consent form to interested and eligible participants in person. Interested and eligible participants provided in-person signed consent after the orientation.	Study staff reviewed the study procedures (including compensation, participation commitment, contacts, and study timelines) and provided the study consent form to interested and eligible participants during a virtual consent session. Interested and eligible participants provided virtual signed consent using approved telehealth technology. A study-materials box was mailed to participants ahead of the virtual orientation session, and all study procedures were reviewed in detail.
Assessment	Medical history and comprehensive demographic information were collected at the consent stage. The ShipShape coordinator or another Health and Wellness Department staff member weighed and measured the participants at the end of baseline, midpoint, and posttreatment sessions, as well as at the 3- and 6-month follow-up visits (if in person). Study staff administered assessment packets at the same time points.	Medical history and comprehensive demographic information were collected as part of the baseline packet. Participants were extensively trained on how to weigh and measure themselves and when to report that information. Study staff collected self-measured weight and other measurements through a Webex (Cisco Inc) private chat at relevant timepoints. Participants brought relevant assessment packets to the virtual intervention sessions, and the 3- and 6-month follow-up packets were completed by mail or telephone. Three brief questions to assess virtual audiovisual quality were asked through Webex polls at the end of each virtual intervention session. The qualitative and quantitative virtual format questionnaire assessed the participants’ experiences with the virtual format.
Intervention	Participants received a binder with an intervention workbook during the first session. Intervention group sizes ranged from 6 to 13 participants. The ShipShape coordinator played recordings of motivational and informational content relevant to ShipShape. ShipShape materials and acceptance and commitment therapy activities were provided live and in person.	Participants received a binder through the mail prior to the start of the study. Intervention group sizes ranged from 4 to 9 participants. Recordings of the ShipShape coordinator and study interventionists were made for the ShipShape and acceptance and commitment therapy content, respectively, and were played during Webex sessions, based on the study arm. Study interventionists facilitated virtual sessions through a combination of live and recorded materials presented during Webex sessions.

#### Recruitment and Screening

Once in-person recruitment was no longer possible, recruitment for participants in the virtual format occurred through the community and by self-referral in response to advertisements within an 80.5-kilometer (50-mile) radius of San Diego. We contracted with BuildClinical LLC, a marketing company that specializes in advertisements for clinical studies through social media. An institutional review board-approved advertisement was posted on various social media platforms (eg, Facebook and Instagram). The advertisement provided brief information on the study and a link to ClinicalTrials.gov. A brief online screening form asked potential participants about their active-duty status, overweight or obesity status, first name, phone number, and the best time for them to receive a phone call. Study staff then contacted potential participants by phone to further determine eligibility.

#### Consent and Assessment

After screened participants were found to be eligible and interested, they scheduled virtual consent and study preparation sessions to review the study in detail, provide signed consent, and review the mailed study materials. Virtual consent and study preparation sessions were held either by phone or Webex (Cisco Inc), and signed consent was obtained using secure telehealth software approved by Veterans Affairs (VA).

The mailed study materials included a binder with comprehensive instructions on all study procedures, which were all completed remotely, and the relevant intervention binder, assessment packets, a digital scale, and other study materials (eg, a tape measure). Study staff provided extensive training to the participants on all at-home measurements, including how to weigh themselves and perform other measurements, prior to virtual intervention sessions 1, 4, and 8. The data were collected privately at the beginning of those sessions. Participants were also instructed to complete their relevant timepoint assessment packets at the beginning (baseline) or end (midpoint and posttreatment) of the intervention sessions. Study staff collected 3- and 6-month follow-up assessments through the mail or by telephone using a protocol that had been used with some of the prepandemic participants.

### Intervention Transition to Virtual Format

#### Virtual Intervention Delivery Protocol

The NMCSD SS coordinator who had primarily led the SS sessions was reassigned to COVID-related duties. Thus, we had to make decisions about how to deliver the intervention materials virtually. We considered providing the materials asynchronously (without live discussion with a facilitator or group of participants) for participants to study at their own pace, allowing more flexibility for participants and a lower burden on facilitators. After discussions with stakeholders, we decided to remain as consistent as possible with the in-person format in terms of the facilitators. We chose to maintain a group format, which allows for the review of materials with a facilitator and opportunities for participants to share experiences and receive support from others. We opted to prepare video recordings of the SS coordinator presenting most of the SS content and a study interventionist presenting the ACT-related content. The virtual group sessions were then held live, with participants viewing the recordings interspersed with live discussion with study interventionists. To maintain live discussion of the SS material, one interventionist acted as the SS facilitator for the nonrecorded content in both the SS and ACT plus SS groups, while another interventionist facilitated the nonrecorded ACT content in the ACT plus SS group. All decisions were guided by the study’s principal investigator.

Video recordings of the SS coordinator (for SS materials) and a study interventionist (for ACT materials) were made with Zoom (Zoom Inc). Recordings were then reviewed, edited, and organized by study staff. Study staff were also present at each session to manage the virtual technology and play the video recordings while the interventionist was focused on delivery of the intervention and attending to group processes. All recordings were reviewed for adherence, competency, content, and logistics by an ACT-trained clinical psychologist. As with the in-person sessions, 20% of the virtual sessions were selected at random and were attended by 2 members of the study team to assign adherence and competency ratings for both the SS and ACT plus SS live interventionists.

#### Session Structure

As with the in-person sessions, participants were required to attend sessions at designated times in the virtual format. Participants received instructions and a list of rules relevant to the virtual format (eg, logging in on time and keeping the camera on at all times). Given that video recordings are inherently less interactive than in-person interactions, the SS and ACT video recordings were followed by interventionist-led live content and discussions. To best support individual behavioral change, some content, such as monitoring hunger level and selecting a personal weekly action plan, was live. Interventionists asked questions about each participant and included interactive content approximately every 30 minutes to encourage engagement. Approximately the same amount of time was spent delivering recorded and live content and group discussions in both conditions.

#### Session Content

Overall, the content of the SS and ACT plus SS interventions remained the same with the transition to the virtual format. The transition from the in-person to the virtual format for the SS condition did not require many modifications, because there were fewer physically interactive activities embedded in the program. The SS coordinator led the in-person sessions with slide show presentations, relevant videos, and discussions. With the transition to the virtual format, these sessions were recorded, with the videos embedded in the recordings.

The transition from in-person to virtual delivery for the ACT plus SS condition required more modifications, because some of the interactive activities were not feasible in the virtual format. The objective was to make modifications to the ACT exercises and metaphors while still targeting the same ACT processes. For example, “tug of war” is an experiential activity that requires a physical prop and at least 2 individuals to demonstrate the ACT processes of acceptance, defusion, and committed action. The study team determined that it was not possible to conduct “tug of war” virtually. This exercise was replaced with “unwanted party guest,” an activity that includes a video and discussion and demonstrates the ACT processes in a similar way. Another example of an activity modification was “cravings and trigger foods.” The study staff brought participant-identified trigger foods to the in-person sessions. For the virtual sessions, participants were instructed to provide their own trigger foods or an image of the food.

### Measures

#### Demographics

Self-reported sociodemographic information was collected at either screening or baseline, including age (in years), sex (male or female), race (American Indian or Alaskan Native; Asian or Pacific Islander; Black or African American; or White, not of Hispanic origin), ethnicity (Hispanic or Latino), paygrade (enlisted or officer), living status (off base or on base), and relationship status (married, partnered, or in a significant relationship).

#### Anthropometrics

In-person measurements were collected by the NMCSD SS coordinator or another member of NMCSD. Weight and height were measured objectively using a stadiometer (Health O Meter 500KL; Pelstar, LLC). BMI was calculated as weight in kilograms divided by height in meters squared. Measurements were made of neck and waist circumference for males, and neck, waist, and hip circumference for females. Study staff calculated body fat percentage with a health and fitness Navy PFA app (Vandersoft, Navy PFA). Virtual participants weighed themselves using a study-provided calibrated digital scale (Body Smart Weight and BMI Digital Scale, Withings SA) and reported their weight and self-reported height to study staff, who calculated BMI. Self-measured neck and waist circumference for males and neck, waist, and hip circumference for females were used by study staff to calculate body fat percentage using the Navy PFA app.

#### Motivation and Confidence

Participants answered the following questions either at screening or baseline: “How motivated are you to participate in a structured weight management/fitness program?” and “How confident are you to complete a structured weight management/fitness program?” Responses to both questions ranged from not at all (0) to extremely (10). Total sum motivation and confidence scores were calculated.

#### Attendance and Participation

Attendance at each of the 8 group sessions was recorded. Participants who completed a minimum of 5 of 8 sessions were predefined as “completers” based on Health and Wellness Department practice. For those who were present at each session, interventionists scored their level of participation as poor (1), adequate (2), or excellent (3). An overall average participation score was calculated.

#### Credibility and Expectations

The 6-item Credibility and Expectations for Improvements (CEI) scale was administered to assess how logical the intervention seemed and how much the participants expected to benefit [12]. Individual items on this measure were transformed to standardized scores (*z* scores) and summed into 2 subscales: credibility and expectancy. Higher scores indicated greater credibility and expectancy.

#### Satisfaction

The 8-item Client Satisfaction Questionnaire (CSQ) was used to measure satisfaction with the interventions [13]. Responses were summed to calculate a total satisfaction score ranging from 0 to 32, with higher scores indicating greater satisfaction.

#### Pragmatics of Technology

To determine if the virtual sessions had adequate audio and video quality, virtual-format participants responded to 3 questions through Webex polls at the end of each virtual session. Questions were asked about audio quality, video quality, and overall satisfaction with the technical aspects of the session, with ratings ranging from very poor (1) to very good (5). An average score was calculated for each item across all participants and visits.

#### Evaluation of Virtual Format

Virtual participants completed an investigator-created virtual format questionnaire (VFQ) with both open-ended and multiple-choice items to examine their perceptions of virtual-format effectiveness after the completion of the intervention. Participants responded to the open-ended question “Overall, what did you find most useful in the course?” and a word cloud map was used to assess the most common words from participants’ responses to this question (Multimedia Appendix 1). Five Likert-type items asked about satisfaction with the virtual format, with responses ranging from strongly dissatisfied (1) to strongly satisfied (5). An additional 4 items asked about the timing and frequency of sessions to assist with the design of future virtual interventions.

### Statistical Analyses

Descriptive statistics were computed to characterize the samples. Data distributions were examined, and independent sample *t* tests (for continuous outcomes) and chi-square tests (for categorical outcomes) were used to compare the characteristics of in-person to virtual participants. Descriptive statistics were used to assess the participants’ experience in the virtual format. A power analysis was not performed, as this was a secondary analysis of already collected data. All statistical tests were 2-tailed. The significance level was set at *P*<.05. Data analysis was performed in SPSS (version 28; IBM, Inc). Qualitative analysis of the open-ended VQF question was conducted using Atlas.ti Word Cloud (version 9, Atlas.ti Inc).

### Ethics Approval

All methods and technology for the virtual format were approved by the Veterans Affairs San Diego Healthcare Systems institutional review board and research and development committee (H160150) and were Health Insurance Portability and Accountability Act–compliant. The original trial was registered on ClinicalTrials.gov (NCT03029507).

## Results

Before the pandemic, a total of 248 potential participants were screened through the NMCSD, of whom 154 (62.1%) were enrolled and randomized to in-person groups. During the pandemic, there were 35 potential participants identified through BuildClinical, of whom 23 (65.7%) were enrolled and randomized to virtual groups. Five participants who had consented through the NMCSD immediately prior to the pandemic restrictions were also included in the virtual groups. The average group size was 10 (SD 2.4; range 6-13) for the in-person groups and 6 (SD 2.2; range 4-9) for the virtual groups.

Overall, there were 178 participants, who were on average 29.7 (SD 6.9) years old, 61.8% (110/178) female, 59.6% (106/178) White, and 28.7% (51/178) Hispanic or Latino. Most participants were enlisted (166/178, 93.3%), lived off base (133/178, 74.7%), and were married, partnered, or reported being in a significant relationship (126/178, 70.8%) (Table 2). The average weight was 94.7 kg (208.7 lbs) for the participants overall, 105.1 kg (231.8 lbs) for males, and 87.6 kg (193.1 lbs) for females; average BMI was 33.1 kg/m^2^ (33.5 kg/m^2^ for males and 32.9 kg/m^2^ for females), which falls in the obese range. Average body fat percentage was 35.6% (27.2% for males and 40.9% for females), which also indicated obesity for both men and women. There were no significant differences between formats in participant characteristics.

On average, participants were highly motivated (mean score 8.1, SD 1.7) to participate and confident (mean score 7.6, SD 1.9) in their ability to complete a weight management program. Of the 149 participants in the in-person format, 123 (82.6%) completed at least 5 of the 8 sessions; 24 of 29 (82.8%) participants in the virtual format completed at least 5 of 8 sessions. Overall, participation was rated as “excellent” by interventionists in both formats, but participants in the virtual format had significantly higher ratings for participation (*P*<.001). The intervention was found to be credible and have adequate expectations for effectiveness across all participants, with no significant differences for credibility (*P*=.1) or expectancy (*P*=.07) across the 2 formats. The average intervention satisfaction score was high (mean score 29.3, SD 3.2), with no significant differences between formats (*P*=.82).

Across all virtual sessions, average ratings for audio quality (mean score 4.57, SD 0.74), visual quality (mean score 4.59, SD 0.76), and overall technical satisfaction (mean score 4.62, SD 0.71) were between “good” and “very good.” Sixteen of the 29 virtual participants completed the VFQ (Table 3). All respondents were “somewhat satisfied” or “strongly satisfied” with the video and live portions of the virtual classes. Most participants used the intervention binders “weekly” or “a few times a week” and reported being “always” or “mostly attentive” to the video recordings. Over half of the respondents reported using the skills learned in class “at least a few times a week” to “daily.” The word cloud generated from the responses of 16 participants to the open-ended question “Overall, what did you find most useful in the course?” identified 110 unique words. The most common 6 words reported were “mindfulness,” “helpful,” “different,” “food,” “binder,” and “class,” depicting the concepts participants found the most useful (Multimedia Appendix 1).

**Table 2:** Characteristics of in-person and virtual participants (N=178).

Characteristics			Total (N=178)		In-person format (N=149)		Virtual format (N=29)
**Sociodemographics**							
	Age, mean (SD) years	29.7 (6.9)		28.7 (6.6)		34.4 (6.6)	
	Female, n (%)	110 (61.8)		94 (63.1)		16 (55.2)	
**Race, n (%)**							
	American Indian or Alaskan Native	8 (4.5)		5 (3.4)		3 (10.3)	
	Asian or Pacific Islander	15 (8.4)		14 (9.4)		1 (3.4)	
	Black or African American	44 (24.7)		39 (26.2)		5 (17.2)	
	White (not of Hispanic origin)	106 (59.6)		86 (57.7)		20 (69)	
	Hispanic/Latino ethnicity	51 (28.7)		40 (26.8)		11 (37.9)	
**Pay grade, n (%)**							
	Enlisted	166 (93.3)		140 (94)		26 (89.7)	
	Officer	12 (6.7)		9 (6)		3 (10.3)	
**Living status, n (%)**							
	Off base	133 (74.7)		108 (72.5)		25 (86.2)	
	On base	45 (25.3)		41 (27.5)		4 (13.8)	
Married, partnered, or in a significant relationship, n (%)			126 (70.8)		106 (71.1)		20 (69)
Navy referral, n (%)			155 (87.1)		149 (100)		6 (20.7)
**Health status^a^, mean (SD)**							
	Weight	209.1 (37.0)		207.5 (36.4)		217.6 (39.8)	
	Body fat percentage	35.6 (8.9)		35.3 (8.6)		37.4 (10.1)	
	BMI	33.2 (3.9)		33.0 (4.0)		34.1 (3.9)	
**Study experience**							
	Motivation, mean (SD) score	8.1 (1.7)		8.2 (1.7)		7.8 (1.6)	
	Confidence, mean (SD) score	7.6 (1.9)		7.7 (1.9)		7.6 (1.7)	
	Attendance, n (%)	147 (82.6)		123 (82.6)		24 (82.8)	
	Participation, mean (SD) score	2.6 (0.5)		2.5 (0.5)		2.9 (0.4)^b^	
	Credibility and Expectations for Improvements Scale, mean (SD) score	–0.12 (4.1)		–0.13 (4.3)		–0.5 (3.5)	
	Credibility, mean (SD) score	–0.70 (1.9)		–0.13 (1.9)		0.20 (1.6)	
	Expectancy, mean (SD) score	–0.02 (2.7)		0.01 (2.8)		–0.20 (2.5)	
	Client Satisfaction Questionnaire, mean (SD) score	29.3 (3.2)		29.6 (3.2)		27.7 (3.0)	

^a^N=148 for in-person group.

^b^*P*<.001.

**Table 3:** Responses to the virtual format questionnaire (N=16).

Questions (range of responses)	Responses				
	1, n (%)	2, n (%)	3, n (%)	4, n (%)	5, n (%)
How satisfied were you with the information provided in the video recordings? (1 to 5: “strongly dissatisfied” to “strongly satisfied”)	0 (0)	0 (0)	0 (0)	10 (63)	6 (38)
How satisfied were you with the live discussion portion of each class? (1 to 5: “strongly dissatisfied” to “strongly satisfied”)	0 (0)	0 (0)	0 (0)	8 (50)	8 (50)
How frequently did you use the binder (packet of written materials) outside of the classroom? (1 to 5: “never” to “daily”)	2 (13)	0 (0)	8 (50)	6 (38)	0 (0)
During the videos, how much of the time were you able to pay attention (not be distracted by family, environment, email, or other things)? (1 to 5: “rarely” to “always”)	0 (0)	1 (6)	0 (0)	11 (69)	4 (25)
If the video recordings were available to you outside of class, how likely would you have been able to watch the videos on your own before class? (1 to 5: “strongly unlikely” to “strongly likely”)	1 (6)	1 (6)	0 (0)	9 (56)	5 (31)
What would be the ideal total amount of time spent watching video recordings during each class? (1 to 5: “10-20 mins,” “20-30 mins,” “30-40 mins,” “40-50 mins,” and “50-60 mins,” respectively)	1 (6)	7 (44)	4 (25)	3 (19)	1 (6)
What would be the ideal class length? (1 to 5: “<30 mins,” “30-60 mins,” “60-90 mins,” “90-120 mins,” and left blank, respectively)	0 (0)	4 (25)	6 (38)	6 (38)	0 (0)
How many classes in a course would be ideal for you? (1 to 5: “1-2 classes,” “3-4 classes,” “4-6 classes,” “6-8 classes,” and “>8 classes,” respectively)	0 (0)	0 (0)	3 (19)	9 (56)	4 (25)
How often have you used the skills learned during this course? (1 to 5: “never” to “daily”)	0 (0)	2 (13)	4 (25)	7 (44)	3 (19)

## Discussion

### Principal Findings

This paper describes the transition of a weight management RCT among active-duty personnel from an in-person format to a virtual format following the onset of the COVID-19 pandemic. Our study adds to the few existing resources [14-17] on adapting recruitment procedures, protocols for study implementation, and lessons learned for transitioning in-person RCTs to virtual delivery. Major changes from the transition to a virtual format included recruitment, modifications to the study procedures, and intervention delivery. These modifications were deemed successful, as we were able to recruit active-duty personnel with similar demographic characteristics and attendance levels and similar indicators of credibility, expectancy, and satisfaction as the in-person format. Additionally, participants in the virtual format provided positive feedback regarding their experiences and study materials.

### Lessons Learned for Recruitment and Logistics

Recruitment and retention of active-duty personnel in interventional or other longitudinal studies is especially challenging for a variety of reasons, such as the need to obtain command approval, restrictions on the use of incentives, transportation difficulties, frequent change in duty station, deployment and military exercises, and retirement or discharge from the service [10]. We found that community-focused advertisements on social media pages (through BuildClinical) were effective for recruitment, screening, and enrollment. Community-based recruitment broadened our efforts to include the region (as opposed to a specific military medical treatment facility) and was more flexible. This strategy allowed us to recruit quickly and at about the same or higher enrollment rate (23/35, 65.7%, vs 154/248, 62.1%) as the in-person recruitment strategy, which is consistent with previous research [18]. Additionally, study procedures were successfully completed remotely. Future research among active-duty personnel may benefit from using a virtual, community-based recruitment approach and remote study procedures to address the mobility and job demands of this population.

### Lessons Learned About Adapting Intervention Protocols

Adapting the SS and ACT plus SS intervention protocols from in-person to virtual delivery took approximately 8 months. This development period ensured that we could integrate prerecorded content with live content while targeting key SS and ACT concepts. Some content was more easily translated to presentation via recordings (eg, mindfulness exercises, stress management, healthy eating behavior, and exercise information) than other content (eg, experiential exercises that relied on live props). This was challenging, and it took some consideration to re-create these experiential experiences virtually while still targeting the key ACT concepts.

In the initial in-person session, clear behavior guidelines were reviewed and provided in writing, including rules such as “Attendance at all 8 sessions is expected.” In the virtual format, guidelines regarding virtual group behavior were developed, provided to participants in their binders, and reviewed in the first session and as needed during the group sessions. For example, virtual guidelines included rules such as “Keep your camera on and have sufficient lighting so you can be seen” and “Unless you are speaking, please mute your audio.” Setting behavioral guidelines early on and reminding participants of these guidelines were critical to the success of the virtual sessions.

### Lessons Learned About Participants’ Experiences in a Virtual Class

Other than significantly higher facilitator-rated participation for the virtual participants, participants in the virtual and in-person sessions were similar in demographic characteristics and study satisfaction levels, which is consistent with previous studies [19]. The higher levels of participation might have been due to the facilitators specifically requesting participation in the virtual format. It is also possible that the smaller group size (ranging from 4 to 9 people) of the virtual sessions allowed for more interaction [20]. Overall, the virtual participants reported good audio and visual quality and few technical difficulties, which is contrary to previous research with virtual interventions [21]. They also reported being satisfied with the video and live portions of each session, were attentive to the video recordings, and used the study and intervention materials (ie, the binder) frequently. We found that 100% of participants were “somewhat satisfied” to “strongly satisfied” with the discussion portions, reinforcing the usefulness of brief live content presentations and discussions. Results from the word cloud reflected critical aspects of SS and ACT, including an emphasis on mindfulness, food, and helpful strategies. This suggests that virtual delivery of the intervention materials was effective, and that they resonated with participants. Together, these findings indicate that the virtual transition was successful, and that SS and ACT plus SS can be delivered virtually for greater reach and accessibility. Future research can focus on a large-scale virtual RCT of ACT among active-duty personnel to determine its efficacy for weight management.

### Limitations

Although the current study provides valuable insight into how to transition from an in-person RCT to a virtual format, there are a few limitations. First, before the pandemic, active-duty personnel were mandated by the Navy to attend SS, whereas after the pandemic, recruitment was from the community. Thus, it is possible that the virtual participants self-selected to participate in the virtual format instead of doing so based on a Navy mandate. Second, the samples of in-person and virtual participants were unequal, with a much smaller number of participants in the virtual groups; this may also have skewed the findings. Third, data collection strategies were heterogeneous and weight and height assessments were self-measured and self-reported in the virtual format, which may have increased bias [22]. Nevertheless, while we found no significant differences between the groups in demographic or anthropometric characteristics, suggesting that participants in the virtual groups were similar to participants in the in-person groups, our quantitative findings should be interpreted with caution. Additional research with larger samples is needed to determine optimal designs for virtual RCTs of active-duty personnel with overweight or obesity.

### Conclusions

During COVID-19, our research team successfully transitioned a weight management RCT with active-duty personnel from an in-person to a virtual format, including aspects of study design such as recruitment, screening, assessments, procedures, and content delivery. The virtual format led to similar levels of satisfaction and attendance as the in-person format. The successful transition of this study to a virtual format provides support for the use of virtual interventions among active-duty personnel. Furthermore, virtual ACT-based weight management and fitness interventions may be a promising approach to increase the accessibility and reach of these interventions among highly mobile populations, such as active-duty personnel.

## Appendix

Multimedia Appendix 1 Word cloud map of the most common words from participants (N=16) in response to the open-ended question “Overall, what did you find most useful in the course?”.

Multimedia Appendix 2 CONSORT-eHEALTH checklist.

## Abbreviations

ACT: acceptance and commitment therapy
CEI: Credibility and Expectations for Improvements
CSQ: Client Satisfaction Questionnaire
NIH: National Institutes of Health
NMCSD: Naval Medical Center San Diego
PFA: Physical Fitness Assessment
RCT: randomized controlled trial
SS: ShipShape
VA: Veterans Affairs
VFQ: virtual format questionnaire
