# Supplementary figures and images for: Lessons Learned From Transition of an In-Person to a Virtual Randomized Controlled Trial for Weight and Fitness Concerns in Active-Duty Service Members: Survey Study
Source: J Med Internet Res. 2022 Nov 10;24(11):e37797. doi: 10.2196/37797 (PMC9693705; doi:10.2196/37797)

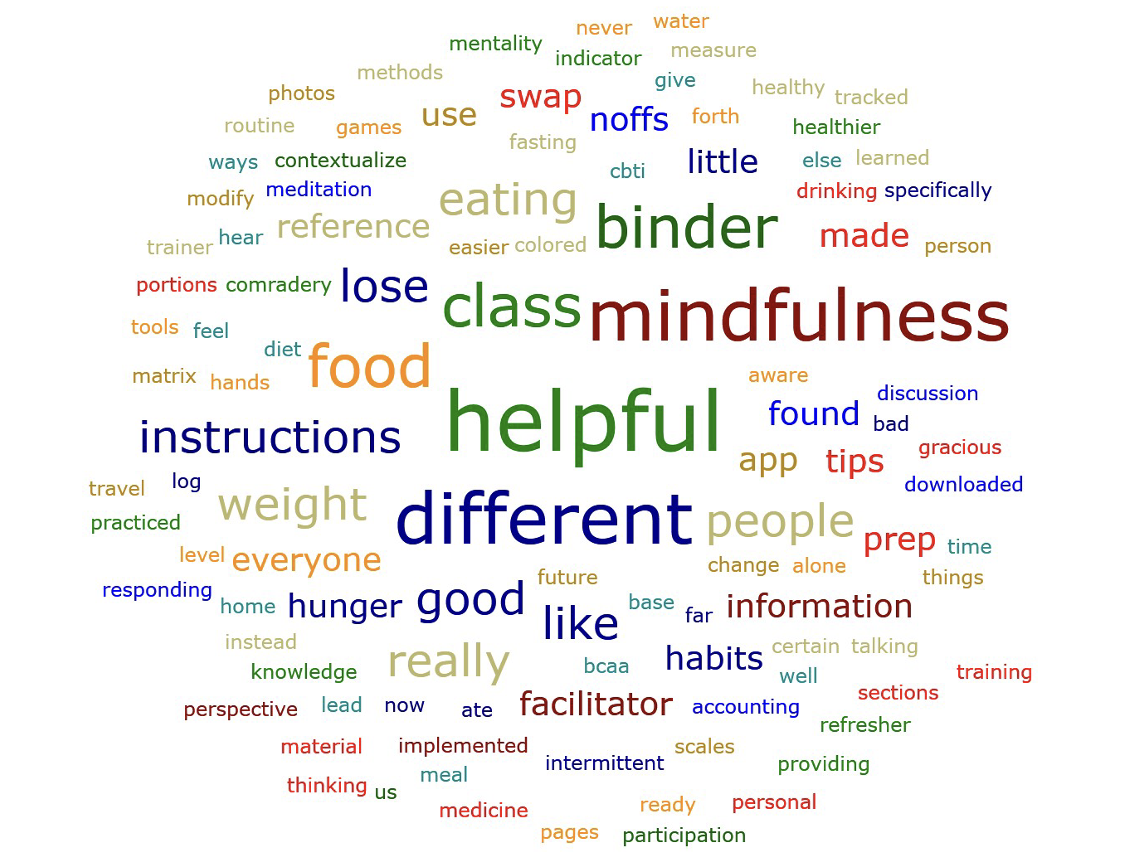

Supplement: Multimedia Appendix 1 [file jmir_v24i11e37797_app1.png]
